# Omega-3 Fatty Acid Supplementation and Cardiovascular Disease Risk: Glass Half Full or Time to Nail the Coffin Shut?

**DOI:** 10.3390/nu10070864

**Published:** 2018-07-04

**Authors:** Kevin C. Maki, Mary R. Dicklin

**Affiliations:** Midwest Biomedical Research, Center for Metabolic and Cardiovascular Health, Glen Ellyn, IL 60137, USA; mdicklin@mbclinicalresearch.com

**Keywords:** omega-3 fatty acids, long-chain polyunsaturated fatty acids, cardiovascular disease, meta-analyses, diet recommendations, cardiac death, coronary heart disease, randomized controlled trials, triglycerides

## Abstract

There has been a great deal of controversy in recent years about the potential role of dietary supplementation with long-chain omega-3 polyunsaturated fatty acids (n-3 PUFA) in the prevention of cardiovascular disease (CVD). Four recent meta-analyses have been published that evaluated randomized, controlled trial (RCT) data from studies that assessed the effects of supplemental n-3 PUFA intake on CVD endpoints. The authors of those reports reached disparate conclusions. This review explores the reasons informed experts have drawn different conclusions from the evidence, and addresses implications for future investigation. Although RCT data accumulated to date have failed to provide unequivocal evidence of CVD risk reduction with n-3 PUFA supplementation, many studies were limited by design issues, including low dosage, no assessment of n-3 status, and absence of a clear biological target or pathophysiologic hypothesis for the intervention. The most promising evidence supports n-3 PUFA supplementation for prevention of cardiac death. Two ongoing trials have enrolled high cardiovascular risk subjects with hypertriglyceridemia and are administering larger dosages of n-3 PUFA than employed in previous RCTs. These are expected to clarify the potential role of long-chain n-3 PUFA supplementation in CVD risk management.

## 1. Introduction

There has been a great deal of controversy in recent years about the role of dietary supplementation with long-chain omega-3 polyunsaturated fatty acids (n-3 PUFA) in the prevention of cardiovascular disease (CVD). The predominant long-chain n-3 PUFAs in the diet are eicosapentaenoic acid (EPA) and docosahexaenoic acid (DHA). Docosapentaenoic acid (DPA) is present in small quantities in the diet and is an intermediate in the conversion of EPA to DHA. The predominant sources of long-chain n-3 PUFAs in the diet are oily fish and other types of seafood. Alpha-linolenic acid is also an essential omega-3 fatty acid that can be obtained through consumption of plant foods such as flax, canola and soybean oils [1]. However, humans convert this shorter chain n-3 PUFA to EPA to a very limited degree [2]. The long-chain n-3 PUFAs play key roles in maintaining normal neurologic and cardiovascular functions [1]. Recommendations from various health authorities around the world suggest consumption of the equivalent of at least 1–2 oily fish meals a week, which provides 250–500 mg/d of EPA + DHA [3,4,5]. Intakes of EPA + DHA in the US and many other developed countries are well below recommended levels [6,7]. Intakes of long-chain n-3 PUFAs well above those recommended for general health (≥3 g/d) have therapeutic applications, such as lowering the circulating triglyceride (TG) concentration to reduce risk for pancreatitis [1].

Results from early observational studies, such as those of Greenland Eskimos whose diets contained large quantities of long-chain n-3 PUFA, suggested that higher intake was associated with lower CVD risk. Subsequently, the results from the Gruppo Italiano per lo Studio della Sopravvivenza nell’Infarto Miocardico (GISSI)-Prevenzione trial, initially published in 1999, showed lower risk of the primary composite endpoint of death, non-fatal myocardial infarction (MI), and stroke in a group of recent survivors of MI randomly assigned to receive treatment with n-3 PUFA (850 mg/d EPA + DHA as ethyl esters) compared with a control group that received no treatment [8]. The trial also had randomization to vitamin E or no vitamin E, which showed no effect on CVD events. In a two-way analysis comparing those receiving and not receiving n-3 PUFA (with or without vitamin E), the incidence of the primary outcome was reduced by 10%, although this appeared to have been driven mainly by 20–26% lower rates of deaths from cardiac causes, which included mainly deaths from acute MI, sudden and arrhythmic deaths, and deaths from heart failure.

In mechanistic and animal studies, long-chain n-3 PUFA supplementation was found to have a number of potentially beneficial actions that included reducing levels of TG and fibrinogen, inhibition of vascular adhesion molecule expression, enhancement of endothelial function and arterial compliance, and inhibition of some pro-inflammatory cytokines [9,10]. Given the biologic plausibility, promising data from observational studies, and the benefit shown in the GISSI-Prevenzione trial, a great deal of enthusiasm was generated for additional studies to evaluate the effects of n-3 PUFA interventions on CVD risk.

To date, results have been reported from more than 30 randomized, controlled trials (RCTs) that reported the effects of long-chain n-3 PUFA supplementation on CVD event outcomes [11]. In addition, at least four RCTs are underway, or have recently been completed, which include long-chain n-3 PUFA interventions and assess CVD events [12,13,14,15]. Four recent meta-analyses have been published that evaluated the available RCT data from studies that assessed the effects of n-3 PUFA intervention on CVD-related endpoints. The authors of these papers reached disparate conclusions regarding the potential efficacy of long-chain n-3 PUFA supplementation for CVD risk reduction [11,16,17,18]. In one case, the authors concluded that there is little reason to continue to study long-chain n-3 PUFA for CVD protection [18], whereas others have suggested that long-chain n-3 PUFA supplementation shows evidence suggestive of benefit, and that additional investigation is clearly warranted [16,17].

After reviewing the available evidence, the American Heart Association released an advisory to update prior guidance regarding clinical use of dietary long-chain n-3 fatty acid supplementation, in which the authors concluded [19]:

“Although recent RCT evidence has raised questions about the benefits of omega-3 supplementation to prevent clinical CVD events, the recommendation for patients with prevalent CHD such as a recent MI remains essentially unchanged: Treatment with omega-3 PUFA supplements is reasonable for these patients. Even a potential modest reduction in CHD mortality (10%) in this clinical population would justify treatment with a relatively safe therapy. We now recommend treatment for patients with prevalent heart failure without preserved left ventricular function to reduce mortality and hospitalizations (9%) on the basis of a single, large RCT”.

In contrast, a Medical News and Perspectives piece published recently in the Journal of the American Medical Association was titled Another Nail in the Coffin for Fish Oil Supplements [20]. The story quotes one of the authors of the meta-analysis cited earlier [18], who stated that their analysis “doesn’t provide any support for the current recommendation from the American Heart Association to use omega-3 fatty acids for the prevention of fatal coronary heart disease or any coronary heart disease in people with prior vascular disease”.

The opinions among experts may thus be characterized as those who view the glass as half full (i.e., the available results are promising and justify further research) and those who believe it is time to nail the coffin shut on the question of potential benefits of long-chain n-3 PUFA supplementation for CVD risk reduction [18,19,20,21]. Our view falls into the glass half full category. Therefore, the objectives of this editorial review are to summarize results from recent meta-analyses of RCT data evaluating the effects of long-chain n-3 interventions on various CVD outcomes and to explain why we are of the opinion that additional research is warranted to evaluate potential cardiovascular benefits of higher dosages of long-chain n-3 PUFAs, particularly for reducing risk of cardiac death in subgroups at high risk.

## 2. Results from Recent Omega-3 Meta-Analyses

Key results from four recent meta-analyses of RCT data from investigations of long-chain n-3 PUFA administration, compared to control or no treatment, are summarized in Table 1 in order to highlight similarities and differences in the reported findings [11,16,17,18]. Alexander et al. included 18 RCTs in their evaluation of effects of long-chain n-3 PUFA interventions on coronary heart disease (CHD) events [16]. A modestly lower (6%) incidence of CHD events was reported for the intervention group, which was not statistically significant. A statistically significant 19% reduction in coronary death was reported for a subset of five trials that reported this outcome.

Maki et al. reported that the pooled relative risk (RR) for cardiac death was 8% lower (*p* = 0.015) for the intervention groups in 14 trials that provided the intervention as a dietary supplement or pharmaceutical n-3 PUFA concentrate [17]. The outcome in this analysis included deaths from CHD, cardiac arrhythmia and heart failure. The primary analysis included secondary prevention and mixed primary and secondary prevention subjects, although trials of subjects with implanted cardiac defibrillators were excluded.

Hooper and colleagues completed a comprehensive meta-analysis for the World Health Organization [11]. Their results indicated that there were no statistically significant effects for cardiovascular (CV) death, CV events, CHD death or stroke, but there was a statistically significant 7% risk reduction in CHD events from a group of 28 RCTs. A separate analysis of stroke incidence showed no evidence of benefit.

In the meta-analysis of 10 RCTs, Aung et al. (the Omega-3 Treatment Trialists’ Collaboration) reported that there were no statistically significant effects of long-chain n-3 PUFA for outcomes of CHD events (nonfatal MI or CHD death), nonfatal MI, stroke, or major vascular events (composite of first occurrence of nonfatal MI or death caused by CHD, nonfatal or fatal stroke, or any revascularization procedure) [18]. However, for CHD death, the pooled effect size showed a marginally significant (*p* = 0.05) 7% reduction in CHD death for the n-3 PUFA intervention groups, an outcome which included sudden cardiac death, deaths due to ventricular arrhythmias and heart failure in patients with CHD, as well as deaths occurring after coronary revascularization or heart transplant.

In addition to the meta-analyses summarized in Table 1, an updated systematic review was prepared for the Agency for Healthcare Research and Quality (AHRQ), U.S. Department of Health and Human Services by Balk et al. [22]. The authors concluded that there was low strength of evidence to support a conclusion of no association between total n-3 fatty acid intake and stroke death or MI, and insufficient evidence for other outcomes. The AHRQ analysis did not include a statistical evaluation of cardiac death because its pre-defined inclusion criteria resulted in examination of just five RCTs reporting cardiac death, and only one that reported cardiac death as a primary outcome.

## 3. Summary of Meta-Analysis Findings by Type of CV Outcome

### 3.1. Stroke and Cardiovascular (CV) Events

The summary above suggests that the current RCT evidence does not support a benefit of the long-chain n-3 PUFA interventions studied on risk for stroke because the two meta-analyses that reported on that outcome showed pooled RRs that indicated non-significantly higher incidence (3% and 6%) in the intervention groups compared with the control groups [11,18]. Because CV events (also termed major vascular events) include both CHD events and stroke, it is not surprising that no benefits were observed for CV events, including CV deaths.

### 3.2. Coronary Heart Disease (CHD) Events

Three of the four meta-analyses reported on the outcome of CHD events. The pooled RRs indicated modestly lower incidence of 6% [16], 7% [11] and 4% [18], but only one of the three had a 95% confidence interval (CI) that did not include the null value of 1.0, indicating statistical significance [4].

### 3.3. Coronary Heart Disease (CHD), Coronary or Cardiac Death

Analyses of deaths from cardiac-related causes were included in all four meta-analyses and three of the four showed significantly lower incidence in the long-chain n-3 PUFA intervention arms. Alexander et al. reported a significant reduction of 19% in coronary deaths, although this analysis pooled data from only five trials [16]. Maki et al. reported a significant reduction of 8% in cardiac death based on data from 14 trials [17]. Hooper et al. reported a non-significant reduction of 7% in CHD death based on pooled data from 21 trials [11], whereas Aung et al. reported a marginally significant reduction of 7% based on data from 10 trials [18].

The summary above indicates that results appear most promising for a potential benefit of long-chain n-3 PUFA supplementation for prevention of cardiac death. As discussed below, such an effect is biologically plausible through mechanisms relating to prevention of arrhythmic events triggered by cardiac ischemia and modulation of adverse cardiac remodeling in response to injury, which has the potential to reduce risks for both heart failure and ventricular arrhythmia [9,19]. However, it is also possible that higher dosages of long-chain n-3 PUFAs may be effective for lowering risks for non-fatal events through anti-inflammatory and anti-atherosclerotic pathways, particularly in some subsets of those at high CV risk, such as individuals with elevated TG and low high-density lipoprotein cholesterol (HDL-C) [17]. A number of limitations exist in the existing evidence base from RCTs of long-chain n-3 interventions evaluating CV outcomes that suggest biologically plausible hypotheses that, in our view, warrant further investigation. Thus, we feel it is premature to suggest that it is time to “nail the coffin shut” regarding a potential role for long-chain n-3 PUFA supplementation as a strategy to reduce CV event risk.

## 4. Mechanisms through Which Long-Chain n-3 Polyunsaturated Fatty Acids (PUFAs) Could Potentially Lower Cardiovascular Disease (CVD) Risk

Mozaffarian and colleagues have reviewed the mechanisms through which increasing n-3 PUFA intake may influence risk for CV events [9,10]. These include metabolic effects, such as reducing plasma levels of TG and TG-rich lipoprotein cholesterol levels; effects on the myocardium, such as reducing susceptibility to ventricular arrhythmia (especially that induced by ischemia); lowering heart rate and myocardial oxygen demand, as well as increasing left ventricular diastolic filling; vascular effects, such as reducing vascular adhesion molecule expression and increasing flow-mediated vasodilation. Additional effects include reducing platelet aggregation and increasing the production of n-3 PUFA metabolites that may reduce inflammation and enhance resolution of inflammatory responses. For many mechanisms, the dose-response relationship is not well defined and potential interactions with baseline n-3 PUFA intake and status have not been extensively studied.

## 5. Limitations of the Current Randomized Controlled Trial (RCT) Evidence Base

The existing RCT evidence base has several limitations, including: low dosages of long-chain n-3 PUFA in most trials, lack of assessment of biomarkers of n-3 PUFA status (and status of other relevant fatty acids such as n-6 PUFA) at baseline or during treatment, and selection of study samples in whom the pathophysiologic basis for predicting a benefit was not clear.

## 6. Low Dosages and Lack of n-3 Polyunsaturated Fatty Acid (PUFA) Status Determination before and during Supplementation

Most RCTs of interventions for CVD prevention are designed to evaluate effects on one or more biomarkers of disease risk, such as circulating cholesterol level, blood pressure, or inflammatory markers, in addition to clinical events. This approach provides a mechanistic link between the effects of the intervention on one or more biomarkers and the observed effect(s) on CVD event risk. Long-chain n-3 PUFA have been shown to affect a variety of biomarkers of CVD event risk. However, with the exception of TG-lowering [23], the dose-response characteristics for effects on biomarkers of risk have not been well defined, thus it is unclear whether large enough dosages were employed to induce physiologic changes required to produce CVD benefits. For example, of the 10 trials included in the meta-analysis by Aung et al., only two tested dosages >1.0 g/d of EPA + DHA and none tested dosages >2.0 g/d [18]. As an analogy, the drug atorvastatin is used to lower CVD risk, primarily through its effect to reduce low-density lipoprotein cholesterol (LDL-C). Beneficial effects have been demonstrated in trials with dosages ranging from 10–80 mg/day [24]. However, if a large majority of trials had been conducted with 2 mg/day of atorvastatin, it is doubtful that a reduction in CVD risk would have been consistently demonstrated because the CVD event risk reduction with statin therapy has been shown to be closely related to the degree of LDL-C lowering, with each mmol/L reduction associated with a 22% reduction in risk [24].

Long-chain n-3 fatty acid incorporation into tissues can be estimated through the evaluation of the levels in blood pools such as plasma phospholipids and erythrocyte membrane phospholipids. Values for these two blood pools are highly correlated with one another and with levels of long-chain n-3 fatty acids in tissue phospholipids, including cardiac tissue [25]. Assessment of levels of EPA and DHA in plasma or tissue pools eliminates many of the measurement issues associated with estimating dietary long-chain n-3 PUFA intakes. Thus, biomarkers of n-3 status may be useful as proxy measures for the adequacy of the dosage employed to impact the various pathways through which CVD benefits might be realized [25,26].

The relationships between total long-chain n-3 PUFA content [EPA + DPA + DHA] of plasma phospholipid fatty acids with selected CV mortality outcomes among subjects in the Cardiovascular Health Study are shown in Table 2 [27]. Significant inverse associations were observed for CV death, CHD death and arrhythmic death, but not for death from stroke. For CV death, CHD death and death due to cardiac arrhythmia, no significant inverse associations with risk were observed in the second and third quintiles compared with the first quintile. Significantly lower risks were observed in the fourth and fifth quintiles for CV and CHD death, and only in the fifth quintile for arrhythmic death. In a separate analysis from the Cardiovascular Health Study, the same investigators evaluated the association between plasma phospholipid n-3 PUFA levels and risk for congestive heart failure. In the full sample, only the top quartile of total plasma phospholipid n-3 PUFA (EPA + DPA + DHA) showed a significant inverse relationship with incident congestive heart failure (hazard ratio [HR] 0.70, 95% CI 0.49–0.99) [28].

Del Gobbo et al. completed a pooled analysis of data from cohort studies that assessed biomarker levels of n-3 fatty acids [29]. They found that each 1-standard deviation (SD) increase in long-chain n-3 fatty acids (EPA + DPA + DHA) in plasma phospholipids was associated with an HR for fatal CHD of 0.88 (95% CI 0.80–0.96). West and colleagues (2018, in press) provided supplements containing an average of 1.26 g/d of EPA + DHA in ethyl ester form. The mean increase in the omega-3 index (erythrocyte phospholipid EPA + DHA) was 1.8%. The median level of supplementation in the 10 RCTs included in the Aung et al. meta-analysis was ~850 mg/d of EPA + DHA, with eight of 10 studies providing the supplements in ethyl ester form [18]. If approximate dose-proportionality is assumed, the results from the study by West et al. suggest that a dosage of 850 mg/d of EPA + DHA as ethyl esters would be expected to increase the omega-3 index by ~1.2% [30]. Harris et al. [26] showed that the SD for estimated omega-3 index in 10 cohorts evaluated in the Del Gobbo et al. [29] analysis was 2.1%. Therefore, the expected median increase in omega-3 index for the RCTs in the Aung meta-analysis was ~0.57 SDs [5]. Accordingly, the expected effect on fatal CHD would be 0.88^0.57^ = 0.93, a 7% reduction in fatal CHD risk. This value is consistent with the rate ratios of 0.93 for CHD death reported by both Aung et al. [18] and Hooper et al. [11], as well as the value of 0.92 for cardiac death reported by Maki et al. [17] (see Table 1). Thus, based on results from prospective cohort studies, it is reasonable to speculate that supplementation with higher dosages of long-chain n-3 PUFA than used in most of the RCTs completed to date might be expected to produce larger reductions in cardiac death (death from CHD, arrhythmia and heart failure). Alexander et al. and Maki et al. reported subgroup analyses for RCTs that employed dosages > or ≥1.0 g/d of EPA + DHA [16,17]. Alexander et al. showed a non-significant pooled RR from seven trials for any fatal CHD event (RR = 0.89, 95% CI 0.58–1.37) [16]. Maki et al. reported a pooled RR for cardiac death of 0.71 (95% CI 0.51–0.99, *p* = 0.043) from seven trials that included 20,418 participants (28% of those in the primary analysis of 14 trials) [17]. Although these subgroup analyses must be interpreted with caution, the results are consistent with the hypothesis that the dosages provided in most of the large-scale RCTs of long-chain n-3 PUFA interventions may have been insufficient to reduce risk for cardiac-related deaths.

Two ongoing trials [Reduction of Cardiovascular Events with EPA-Intervention Trial (REDUCE-IT) and Outcomes Study to Assess Statin Residual Risk Reduction with Epanova in High CV Risk Patients with Hypertriglyceridemia [STRENGTH]) are using dosages of EPA + DHA ≥3 g/d that are well above the ~850 mg/d used in a majority of the largest trials completed to date [12,13]. Two other trials [The Vitamin D and Omega-3 Trial (VITAL) and A Study of Cardiovascular Events in Diabetes (ASCEND)] that have recently been completed and are expected to report results this year used dosages of 840 mg/d of EPA + DHA as ethyl esters [14,15].

## 7. Lack of a Clear Pathophysiologic Hypothesis in Most Trials

A large majority of the trials included in the four meta-analyses summarized in Table 1 were not designed to enroll a group with increased risk attributable to one or more pathophysiological states that could be mitigated by long-chain n-3 supplementation, such as subjects with elevated TG or low omega-3 index. In the future, it would be of interest to conduct an RCT in subjects at high CV risk who have a low omega-3 index, such as individuals with a value <4% (roughly 1-SD below the population mean) [26]. Based on data from observational studies, Harris and von Schacky have suggested a target level of omega-3 index of ≥8.0% for lowering CHD risk (roughly 1-SD above the population mean) [25]. Raising the mean omega-3 index to ≥8.0% from a baseline <4% would likely require a supplemental EPA + DHA ethyl ester dosage of at least 3 g/d [26,30]. If the 12% reduction in risk for each SD increase in omega-3 index based on the relationship observed in cohort studies that measured plasma phospholipid long-chain n-3 fatty acids were to hold true [29], an increase in the omega-3 index from 3.8% to 8.5% would represent an increase of 2.2 SDs (8.5% − 3.8% = 4.7% and 4.7%/2.1% = 2.2 SDs). This would be predicted to reduce risk of CHD death by 1 − 0.88^2.2^ = 0.25 or 25%. Ideally, such a study would include measurements to document the impacts of the intervention on biomarkers of CV risk such as indicators of chronic inflammation, hemodynamic status (e.g., blood pressure and heart rate), endothelial function, lipoprotein lipids and particles, and hemostatic variables. This would allow exploration of the relative importance of various hypothesized mechanisms if a benefit was observed.

To test the hypothesis that long-chain n-3 PUFA supplementation reduces CV risk through pathways related to effects on inflammation and resolution, a study sample could be identified with elevated levels of a biomarker of inflammation, such as high-sensitivity C-reactive protein. The sample could then be randomized to receive long-chain n-3 PUFA or placebo along with other standard therapies. A similar approach was taken in the Canakinumab Anti-inflammatory Thrombosis Outcomes Study (CANTOS) with the monoclonal antibody canakinumab, an agent that inhibits the inflammatory cytokine interleukin-1. CANTOS provided the first direct evidence that an anti-inflammatory intervention could reduce CV event risk [31].

Inflammatory pathways are believed to play a key role in adverse cardiac remodeling [32,33,34,35]. Results from the Omega-3 Acid Ethyl Esters on Left Ventricular Remodeling After Acute Myocardial Infarction (OMEGA-REMODEL) trial support the biologic plausibility of increasing long-chain n-3 PUFA status as a method to reduce adverse cardiac remodeling [36]. After injury, such as an acute MI, chronically elevated cardiac pre-load and/or afterload, or chronic uremia such as occurs in end-stage renal disease, adverse left ventricular remodeling can occur, which can contribute to risks for progressive cardiac dysfunction, heart failure and ventricular arrhythmia [32,33,37]. In OMEGA-REMODEL, subjects with a recent history of MI were randomly assigned to 4 g/d n-3 acid ethyl ester capsules (3.4 g/d EPA + DHA) or placebo, while receiving current guideline-based background therapy [36]. Long-chain n-3 PUFA supplementation significantly reduced left ventricular systolic volume index (the primary outcome variable) as well as non-infarct myocardial fibrosis. Furthermore, a change in the omega-3 index during treatment was related to percent improvement in left ventricular systolic volume index, demonstrating a clear dose-response relationship (*p* for linear trend across quartile of change in omega-3 index < 0.0001). Long-chain n-3 PUFA supplementation was also associated with a significant reduction compared with placebo in the ST2 cardiac stress biomarker. ST2 is a member of the interleukin-1 receptor family that signals the presence and severity of cardiac stress and adverse cardiac remodeling [38]. ST2 elevation provides prognostic information for cardiac mortality post-MI and in heart failure that is independent of other cardiac biomarkers [38]. Therefore, a reduction in ST2 with long-chain n-3 PUFA supplementation in post-MI patients is consistent with the potential to reduce risk of cardiac death, especially because this was accompanied by improved left ventricular end systolic volume and reduced non-infarct cardiac fibrosis. These findings suggest further investigation of the effects of relatively high dosages of long-chain n-3 PUFA is warranted to evaluate the potential for reducing risk for cardiac death in high-risk subsets, such as those with recent MI or left ventricular hypertrophy, individuals with heart failure, and patients with end-stage renal disease.

## 8. Ongoing and Recently Completed Randomized Controlled Trials (RCTs) of Long-Chain n-3 Polyunsaturated Fatty Acid (PUFA) Interventions

There are four ongoing or recently completed CVD outcomes trials testing long-chain n-3 PUFA interventions: REDUCE-IT [12], STRENGTH [13], ASCEND [15] and VITAL [14]. Results from REDUCE-IT, ASCEND and VITAL are due to be released in 2018. Of the trials ongoing or recently completed, two (REDUCE-IT and STRENGTH) are testing the efficacy of higher dosages of long-chain n-3 PUFAs for lowering CV event risk in subjects at high CV risk who have persistent hypertriglyceridemia despite statin therapy. These trials are notable because they are evaluating the effects of treatment on a specific target (hypertriglyceridemia and related lipoprotein abnormalities) in subjects with elevated risk that is believed to be at least partly attributable to an elevated level of the target of the intervention. These studies are also using much larger dosages of EPA and DHA (≥3000 mg/d) than most prior trials. The dosages used in these RCTs should be sufficient to produce clinically meaningful changes in the intervention target (TG) and possibly in other relevant variables such as markers of inflammation, heart rate and blood pressure.

REDUCE-IT enrolled statin-treated patients at elevated CV risk with TG ≥150 mg/dL and <500 mg/dL (but later raised the lower TG requirement to ≥200 mg/dL to increase enrollment of patients with more significant TG elevations) and LDL-C >40 mg/dL and ≤100 mg/dL. The primary outcome is the composite of CV death, nonfatal MI, nonfatal stroke, coronary revascularization or unstable angina. With just over 8000 subjects, a potential concern is that the study may be underpowered to detect an effect in the primary outcome variable. A substantial fraction of the subjects enrolled had TG <200 mg/dL (prior to the change in entry criteria) and subjects were not required to have low HDL-C.

In a meta-analysis investigating the results from RCTs evaluating the effects of agents that primarily lower TG (6 trials of fibrates, 2 of niacin, 1 of a fibrate plus niacin, and 1 of EPA ethyl esters), the pooled CV event risk reduction was 12%, but was higher for the subgroups with elevated TG (18%) and highest (29%) for those with elevated TG plus low HDL-C [39]. Since REDUCE-IT is utilizing a higher dosage of EPA only (3.7 g/d) and is testing a clinically relevant hypothesis in higher risk subjects, we are cautiously optimistic about the potential to demonstrate benefit. Also, the trial has undergone periodic review and has not been stopped for futility, which suggests that some degree of difference in event rates between groups is likely.

Of the four ongoing or recently completed studies, we feel that STRENGTH shows the most promise, based on the higher dosage of EPA + DHA being administered (3.0 g/d EPA + DHA), the form of n-3 used (carboxylic acids, which are better absorbed than n-3 ethyl esters) [40], and the study sample that has been shown in prior subgroup analyses to have the most benefit from TG-lowering medications (i.e., those with high TG and low HDL-C) [13,39]. STRENGTH enrolled patients at high CVD risk with LDL-C < 100 mg/dL, TG ≥ 180 mg/dL and <500 mg/dL and HDL-C < 42 mg/dL for men and <47 mg/dL for women. The primary outcome is the first occurrence of any major adverse cardiac event (including CV death, nonfatal MI, nonfatal stroke, emergent/elective coronary revascularization, or hospitalization for unstable angina). Results are expected in 2020.

Unfortunately, VITAL and ASCEND are each testing a relatively low dosage of 840 mg/d EPA + DHA as n-3 acid ethyl esters. This is approximately the same dosage that has been used in a majority of the trials conducted to date. Therefore, it would not be surprising if these trials produced neutral results.

## 9. Conclusions

RCTs have failed to produce unequivocal evidence of CVD risk reduction with n-3 PUFA supplementation. The evidence appears most promising for prevention of cardiac death, which includes death from ischemic cardiac events, arrhythmia and heart failure. As described herein, the RCTs conducted to date have been limited by design issues, particularly low dosages in many studies, lack of assessment of long-chain n-3 PUFA status prior to and during treatment, and absence of a clear biological target for the intervention in many studies. Two trials (REDUCE-IT and STRENGTH) have enrolled higher CV risk subjects with hypertriglyceridemia and are administering larger dosages of n-3 PUFA (≥3.0 g/d EPA + DHA) than used in most prior RCTs. These trials have addressed many of the design limitations in prior studies; therefore, we view them as the most likely to clarify the potential role of long-chain n-3 PUFA supplementation in CVD risk management.

## Figures and Tables

**Table 1:** Characteristics and selected results from four recent meta-analyses of data from randomized controlled trials of long-chain omega-3 polyunsaturated fatty acid administration compared to control or no treatment.

Meta-Analysis Reference	Trials Included (n)	Subjects Included (n)	Outcomes Evaluated	Pooled Effect Size (Risk Ratio, 95% CI)
Alexander, et al. [16]	18	93,633	CHD event (combination of fatal or nonfatal MI, coronary death, sudden cardiac death, angina)	0.94 (0.85–1.05)
	5	41,350	Coronary death (fatal MI, death from other acute or subacute forms of CHD, or death from chronic CHD)	0.81 (0.65–1.00) *
Maki, et al. [17]	14	71,899	Cardiac death (death from CHD, cardiac arrhythmia, or heart failure)	0.92 (0.86–0.98) *
Hooper, et al. [11]	25	67,772	CV death (death from any CV cause; death from individual CV causes were summed if no report of CV death; cardiac death used if CV death not reported)	0.95 (0.87–1.03)
	32	89,362	CV event (non-fatal MI, CHD death, fatal and non-fatal stroke) ^1^	0.99 (0.94–1.04)
	21	73,491	CHD death (coronary death or, when not reported, ischemic heart disease death, fatal MI or cardiac death)	0.93 (0.79–1.09)
	28	84,301	CHD event (CHD or coronary event, total MI, acute coronary syndrome or stable or unstable angina)	0.93 (0.88–0.97) *
	28	89,358	Stroke (fatal and non-fatal stroke, hemorrhagic and ischemic)	1.06 (0.96–1.16)
Aung, et al. [18]	10	77,917	CHD event (nonfatal MI, CHD death)	0.96 (0.90–1.01)
	10	77,917	Nonfatal MI	0.97 (0.89–1.05)
	10	77,917	CHD death (sudden cardiac death, deaths due to ventricular arrhythmias and heart failure in patients with CHD, MI or deaths occurring after coronary revascularization or heart transplant)	0.93 (0.85–1.00) *
	10	77,917	Stroke (ischemic, hemorrhagic, unclassified/other)	1.03 (0.93–1.13)
	10	77,917	Major vascular event (composite of first occurrence of nonfatal MI or death caused by CHD, nonfatal or fatal stroke, any revascularization procedure)	0.97 (0.93–1.01)

Abbreviations: CHD, coronary heart disease; CI, confidence interval; CV, cardiovascular; CVD, cardiovascular disease; MI, myocardial infarction. ^1^ The paper did not provide a discrete definition of CV event, but an examination of the studies included suggests these were the types of CV events considered in the calculation. * Indicates pooled relative risk estimate with a *p* value of ≤0.05.

**Table 2:** Multivariate-adjusted associations between plasma phospholipid total long-chain omega-3 fatty acid (EPA + DPA + DHA) quintiles with incident mortality from selected cardiovascular causes [27].

Type of Event	Q1	Q2	Q3	Q4	Q5	*p* for Trend
CV Death	1.00	0.92 (0.72–1.19)	1.05 (0.82–1.35)	0.74 (0.56–0.98)	0.65 (0.48–0.87)	<0.001
CHD Death	1.00	0.88 (0.64–1.22)	1.03 (0.75–1.41)	0.62 (0.43–0.89)	0.60 (0.42–0.87)	0.002
Arrhythmic Deaths	1.00	0.79 (0.50–1.24)	1.07 (0.70–1.63)	0.68 (0.42–1.10)	0.52 (0.31–0.86)	0.008
Stroke Death	1.00	0.92 (0.53–1.58)	1.11 (0.66–1.88)	0.84 (0.48–1.48)	0.60 (0.32–1.12)	0.092

Abbreviations: CHD, coronary heart disease; CV, cardiovascular; DHA, docosahexaenoic acid; DPA, docosapentaenoic acid; EPA, eicosapentaenoic acid; Q, quintile.
